# Supplementary material for: Cell division angle regulates the tissue mechanics and tunes the amount of cerebellar folding
Source: bioRxiv. 2023 Jul 21:2023.07.21.549165. Preprint. [Version 1] doi: 10.1101/2023.07.21.549165 (PMC10370211; doi:10.1101/2023.07.21.549165)
Supplement: Supplement 1 [file NIHPP2023.07.21.549165v1-supplement-1.pdf]

bioRxiv preprint doi: https://doi.org/10.1101/2023.07.21.549165; this version posted July 21, 2023. The copyright holder for this preprint (which was not certified by peer review) is the author/funder, who has granted bioRxiv a license to display the preprint in perpetuity. It is made available under aCC-BY 4.0 International license.

Supplemental Figure 1: C57Bl/6J and FVB/NJ cerebella at P28 have robustly different levels of folding at the midline of the vermis. A) Sagittal midline sections of 5 C57Bl/6J cerebella **B)** C57Bl6/J cerebella showing heterotopia between lobule 8 and Lobule 9. **C)** Sagittal midline sections of 5 FVB/NJ. All cerebella were stained with H&E. Scale Bars: 0.5 mm.

Supplemental Figure 2: Size difference between C57Bl/6J and FVB/NJ is regionally regulated. A) Positive curvature. P-value reported. **B)** Lobule lengths. All regions statistically different (one-way ANOVA p-value reported) except for Lobule 8. Two-way ANOVA (lobules and strains) interaction p-value: 7.68e-20. **C)** Lobule lengths as a percentage of total length. Brackets indicate statistical differences (one-way ANOVA p-value reported). Two-way ANOVA (lobules and strains) interaction p-value: 0.00e+00. **D)** Image showing anterior (blue shading) and posterior (yellow shading) regions of cerebellum. **E)** Folding index of anterior and posterior regions. Brackets indicate statistical differences. One-way ANOVA p-value reported. Two-way ANOVA (Regions and strains) interaction p-value: 0.048. For full statistics see statistics table. (mean s.d.)

Supplemental Figure 3: Global ratio of growth diverges during the critical period. A) Residuals from fitting each strain to individual Gompertz function (see Fig. 2A). **B)** Residuals are poorly patterned and larger when data from Fig. 2A is combined and fitted to one Gompertz function. **C-E)** The three parameters of the individual Gompertz function fittings are distinct between the strains. **F-H)** At E16.5 the area (p-value 0.7353), length (p-value: 0.9473), and the ratio of the area and length (p-value 0.417) are unchanged between the strains. **I)** Growth ratio from the start (E16.5) to 1mm2 (~P0). Multiple linear regression analysis interaction p-value reported showing no difference between the slopes. **J)** Residuals are small and well patterned with a single combined fit. **K,L)** The parameters of the individual fittings are overlapping showing no difference between the strains at this early period of growth. For full statistics see statistics table. (mean s.d.)

Supplemental Figure 4: Lobule growth ratios are higher in FVB/NJ than in C57Bl/6J and the resulting differential-expansion is dependent of the geometry of the lobule. A) Multiple linear regression analysis of L4-5 region. Interaction p-value reported. Showing difference in slopes. R-squared values reported for individual fits. **B)** Multiple linear regression analysis of L8 region. Interaction p-value reported. Showing difference in slopes. R-squared values reported for individual fits. **C-D)** Multiple linear regression analysis within each stain. Interaction terms for differences between L6-7 and L4-5 or L8 reported. **E)** Calculated slope parameters from linear regression analysis of L4-5, L6-7, and L8 for both strains showing that the slopes are all increased in FVB/NJ compared to C57Bl/6J and the greatest increase is in L6-7. **F)** Cartoon depicting balanced growth ratio (length/Area) curves for common 2-D shapes. **G)** The residuals calculated from the predictive growth curves show that the growth ratio of C57Bl/6J is more similar to its balanced growth curve than FVB/NJ. **H,I)** L6-7 has a slight difference in geometry between the strains with C57Bl/6J having more length per area than FVB/NJ. P-value reported. (mean s.d.)

bioRxiv preprint doi: https://doi.org/10.1101/2023.07.21.549165; this version posted July 21, 2023. The copyright holder for this preprint (which was not certified by peer review) is the author/funder, who has granted bioRxiv a license to display the preprint in perpetuity. It is made available under aCC-BY 4.0 International license.

Supplemental Figure 5: Lobule geometry regionally regulates folding amount. A) Sagittal midline sections of L8 region stained with Dapi at the start and end of the critical period in FVB/NJ and C57Bl/6J showing the constraints from the surrounding lobule regions and the limited exposed surface Scale bar: 200µm **B)** Model of Lobule 8 expansion. The width of the lobule which sets the parameters for the semi-circle is fixed while the length is allowed to expand. **C)** Balanced expansion curves for such constrained shapes are linear and the slope decreases as the fixed width is increased. **D)** The growth ratio of L8 is well predicted in C57Bl/6J and FVB/NJ by this type of constrained growth showing no evidence of differential-expansion. **E)** The folding index shows that L8 in both strains remains unfolded as its growth ratios remain balanced. **F)** Sagittal midline sections of L45 stained with Dapi at the end of the critical period. Cyan line: EGL length. White line: Positive curvature. Scale bar: 200µm **G)** Folding index of L45 through the critical period. While L4-5 in C57Bl/6 remains unfolded the measured increase in folding index comes from the complex shape of the lobule region.

Supplemental Figure 6: EGL thickness is regionally varied within the cerebellum and correlates with folding wavelength. A) EGL thickness at end of critical period. Brackets indicate regions with statistical difference. One-way ANOVA p-value reported. Two-way ANOVA interaction (strain and lobule) p-value: 0.00. **B)** Midline sagittal sections of C57Bl/6J and FVB/NJ L6-7 stained with Dapi. Cyan line: covered EGL. White line: exposed EGL surface. **C)** EGL density in exposed surface. One-way ANOVA p-value: 0.2534. **D)** Midline sagittal section of FVB/NJ at P28 showing landmarks placed at the conserved anchoring centers. Black lines show lobule wavelengths. White bracket indicates L7 wavelength. Scale bar: 0.5mm **E,F)** Individual Procrustes alignments of landmarks of C57Bl/6J and FVB/NJ bracket indicates L7 wavelength. **G)** Wavelengths from Procrustes alignment for each lobule region. Brackets indicate statistical differences. One-way ANOVA p-value reported. Two-way ANOVA interaction (strains and lobules) p-value: 1.71e-23. **H)** Wavelengths from real distances. Brackets indicate statistical differences. One-way ANOVA p-value reported. Two-way ANOVA interaction (strains and lobules) p-value: 0.00 **I)** EGL thickness in L6-7-8 region at P0 and P1 for FVB/NJ and C57Bl/6J. p-value reported. (mean s.d.)

Supplemental Figure 7: Purkinje cell density is regionally regulated in the Cerebellum and different between the strains. A) Number of Purkinje cells per lobule at P28. Brackets indicate statistical differences. One-way ANOVA p-value reported. Two-way ANOVA interaction (strains and lobules) p-value 0.00 **B)** Purkinje cell density at P28. C57Bl/6J has higher density of Purkinje cells even L6 that has a reduced number compared to FVB/NJ. Brackets indicate statistical differences. One-way ANOVA p-value reported. Two-way ANOVA interaction (strains and lobules) p-value: 0.2054 indicating that while both have different densities, they have the same pattern of density. **C)** Sagittal midline section of FVB/NJ at P28 stained with Calbindin and Dapi to mark the Purkinje cells. Scale bar: 50µm **D,E)** Purkinje cell density during the critical period in L4-5, L6-7, and L8. Brackets indicate statistical differences. ns = not statistically significant. One-way ANOVA p-values reported. **F,G)** Purkinje cell density during the critical period as a multiple of final density at P28. Brackets indicate statistical differences. One-way ANOVA p-values reported. (mean s.d.)

bioRxiv preprint doi: https://doi.org/10.1101/2023.07.21.549165; this version posted July 21, 2023. The copyright holder for this preprint (which was not certified by peer review) is the author/funder, who has granted bioRxiv a license to display the preprint in perpetuity. It is made available under aCC-BY 4.0 International license.

Supplemental Figure 8: Cell Division Angle is regionally adjusted during the critical period. A-C) Polar plot of cell division angles measured in L4-5, L6-7, and L8 at the start and end of the critical periods. At the end of the critical period the difference between the strains is mostly contained to L6-7. **D)** Combined cell division angles measured from L4-5, L6-7, and L8. **E)** Cell division angle ratio of combined data (D). One-way ANOVA p-value reported. Bracket indicates statistical difference. (mean s.d.)

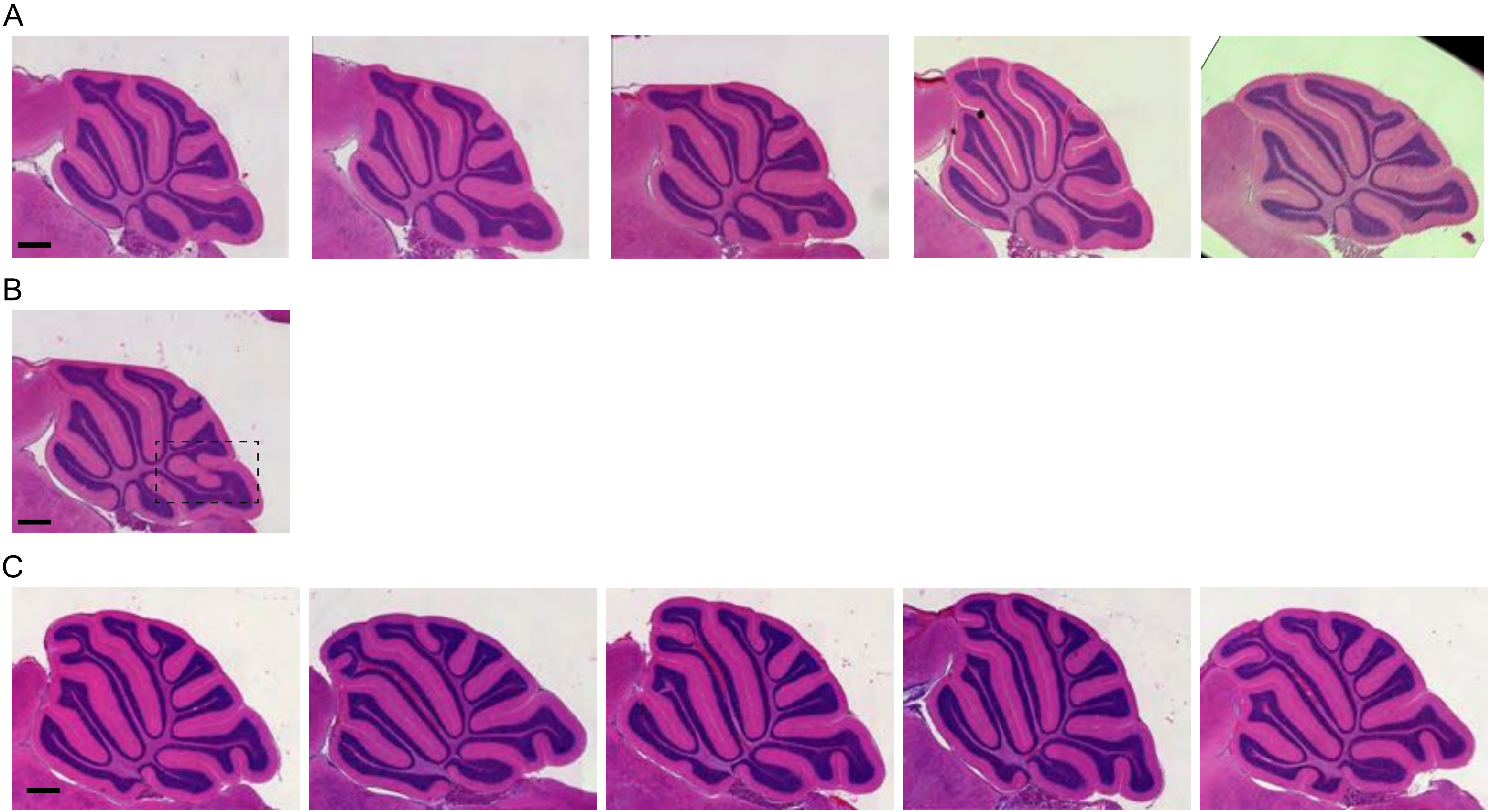
A
B
C

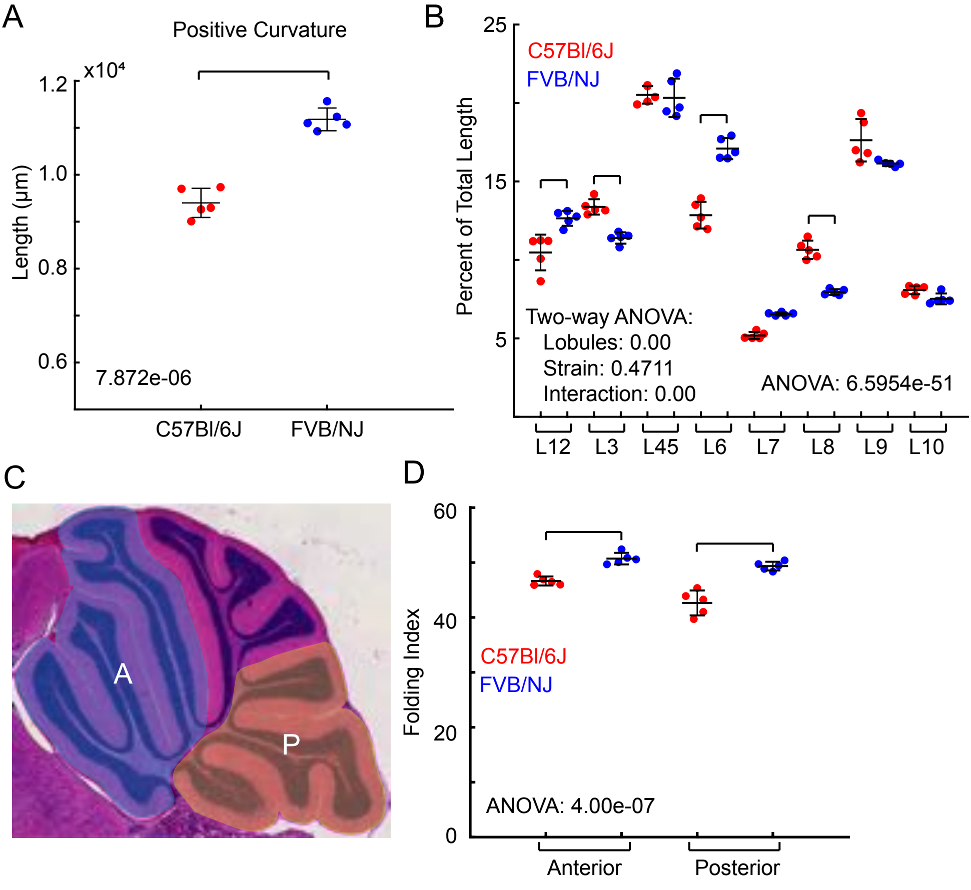
A
Positive Curvature
x10⁴
1.2
1.0
0.8
0.6
Length (µm)
7.872e-06
C57Bl/6J
FVB/NJ
B
25
15
5
Percent of Total Length
C57Bl/6J
FVB/NJ
Two-way ANOVA:
Lobules: 0.00
Strain: 0.4711
Interaction: 0.00
ANOVA: 6.5954e-51
L12
L3
L45
L6
L7
L8
L9
L10
C
A
P
D
60
40
20
0
Folding Index
C57Bl/6J
FVB/NJ
ANOVA: 4.00e-07
Anterior
Posterior

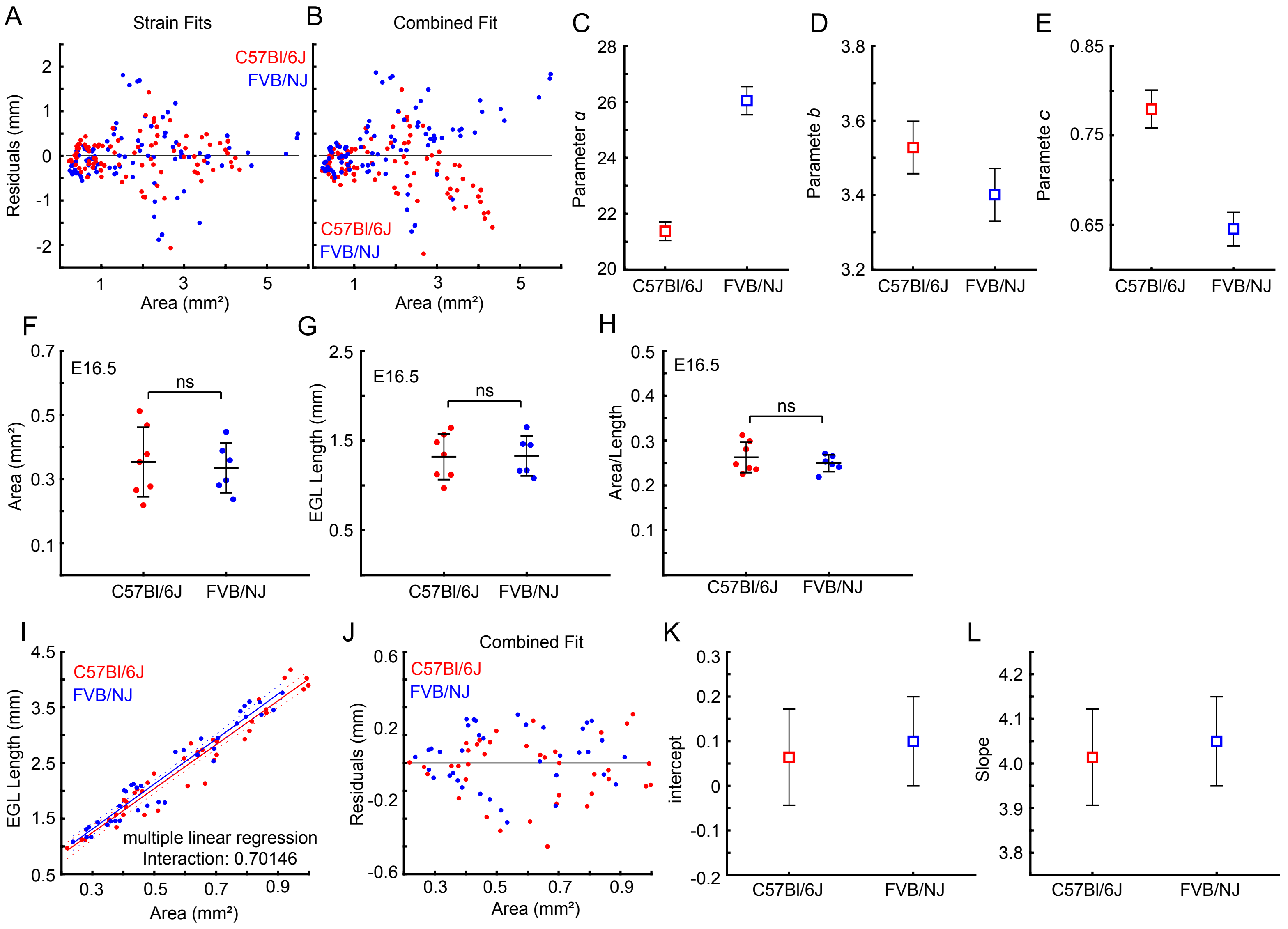
A
Strain Fits
C57Bl/6J
FVB/NJ
Residuals (mm)
Area (mm²)
B
Combined Fit
C57Bl/6J
FVB/NJ
Area (mm²)
C
Parameter a
C57Bl/6J
FVB/NJ
D
Paramete b
C57Bl/6J
FVB/NJ
E
Paramete c
C57Bl/6J
FVB/NJ
F
E16.5
ns
Area (mm²)
C57Bl/6J
FVB/NJ
G
E16.5
ns
EGL Length (mm)
C57Bl/6J
FVB/NJ
H
E16.5
ns
Area/Length
C57Bl/6J
FVB/NJ
I
C57Bl/6J
FVB/NJ
EGL Length (mm)
multiple linear regression
Interaction: 0.70146
Area (mm²)
J
Combined Fit
C57Bl/6J
FVB/NJ
Residuals (mm)
Area (mm²)
K
intercept
C57Bl/6J
FVB/NJ
L
Slope
C57Bl/6J
FVB/NJ

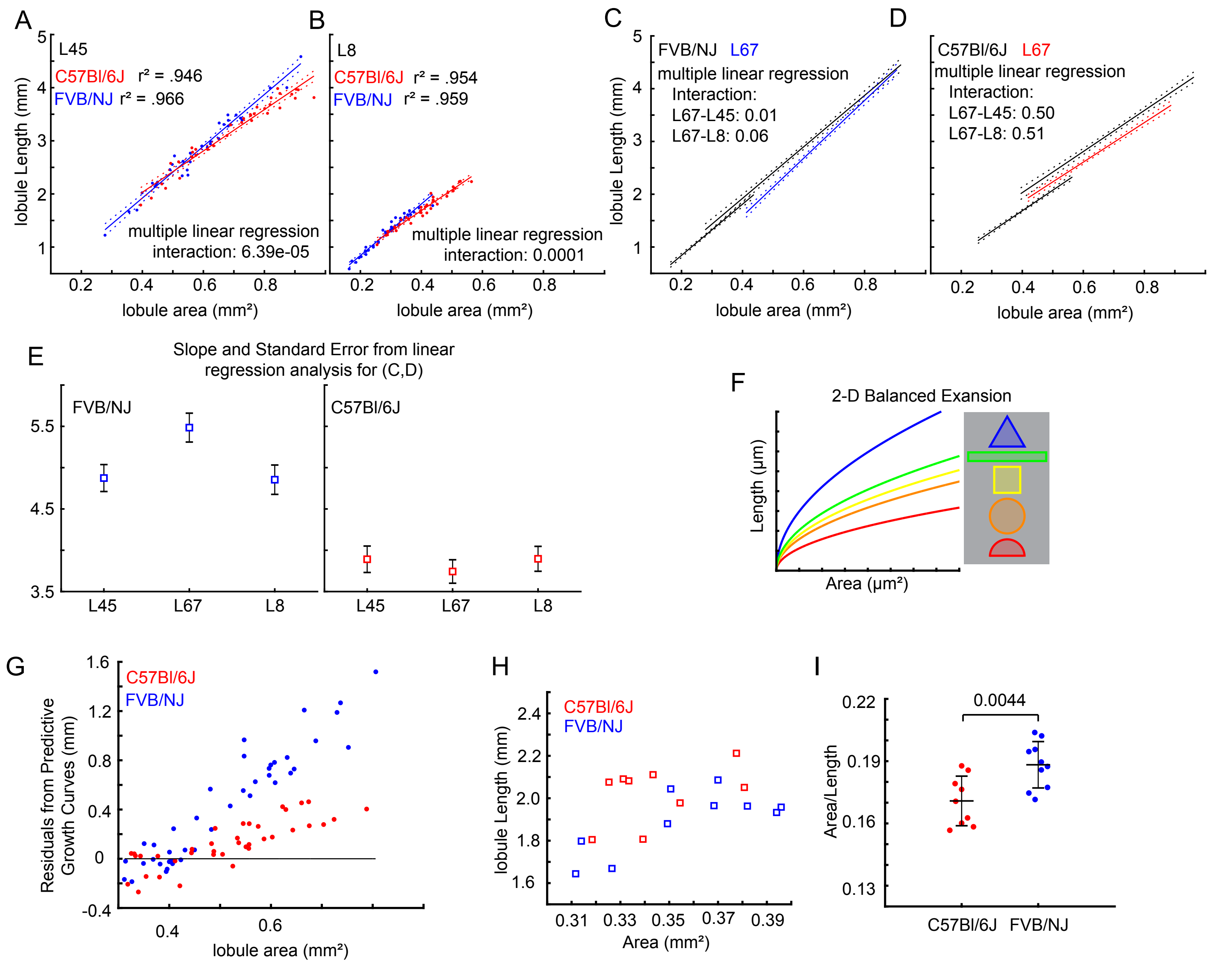
A
L45
C57Bl/6J r² = .946
FVB/NJ r² = .966
multiple linear regression
interaction: 6.39e-05
lobule Length (mm)
lobule area (mm²)
B
L8
C57Bl/6J r² = .954
FVB/NJ r² = .959
multiple linear regression
interaction: 0.0001
lobule area (mm²)
C
FVB/NJ L67
multiple linear regression
Interaction:
L67-L45: 0.01
L67-L8: 0.06
lobule Length (mm)
lobule area (mm²)
D
C57Bl/6J L67
multiple linear regression
Interaction:
L67-L45: 0.50
L67-L8: 0.51
lobule area (mm²)
E
Slope and Standard Error from linear regression analysis for (C,D)
FVB/NJ
C57Bl/6J
L45 L67 L8
F
2-D Balanced Exansion
Length (µm)
Area (µm²)
G
C57Bl/6J
FVB/NJ
Residuals from Predictive Growth Curves (mm)
lobule area (mm²)
H
C57Bl/6J
FVB/NJ
lobule Length (mm)
Area (mm²)
I
0.0044
Area/Length
C57Bl/6J FVB/NJ

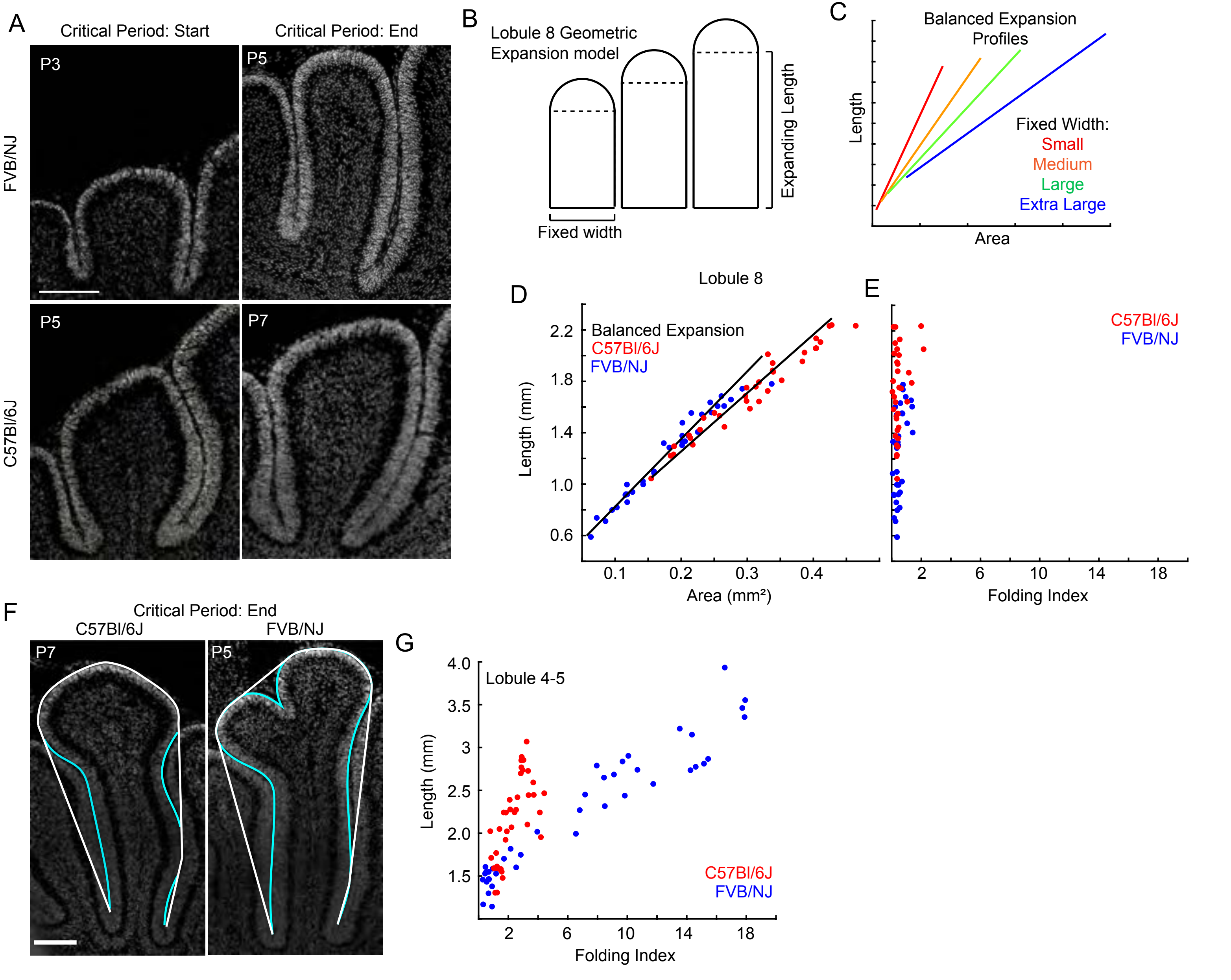

A
Critical Period: Start
Critical Period: End
FVB/NJ
C57Bl/6J
P3
P5
P5
P7
B
Lobule 8 Geometric Expansion model
Expanding Length
Fixed width
C
Balanced Expansion Profiles
Length
Area
Fixed Width:
Small
Medium
Large
Extra Large
Lobule 8
D
Balanced Expansion
C57Bl/6J
FVB/NJ
Length (mm)
Area (mm²)
E
C57Bl/6J
FVB/NJ
Folding Index
F
Critical Period: End
C57Bl/6J
FVB/NJ
P7
P5
G
Lobule 4-5
Length (mm)
Folding Index
C57Bl/6J
FVB/NJ

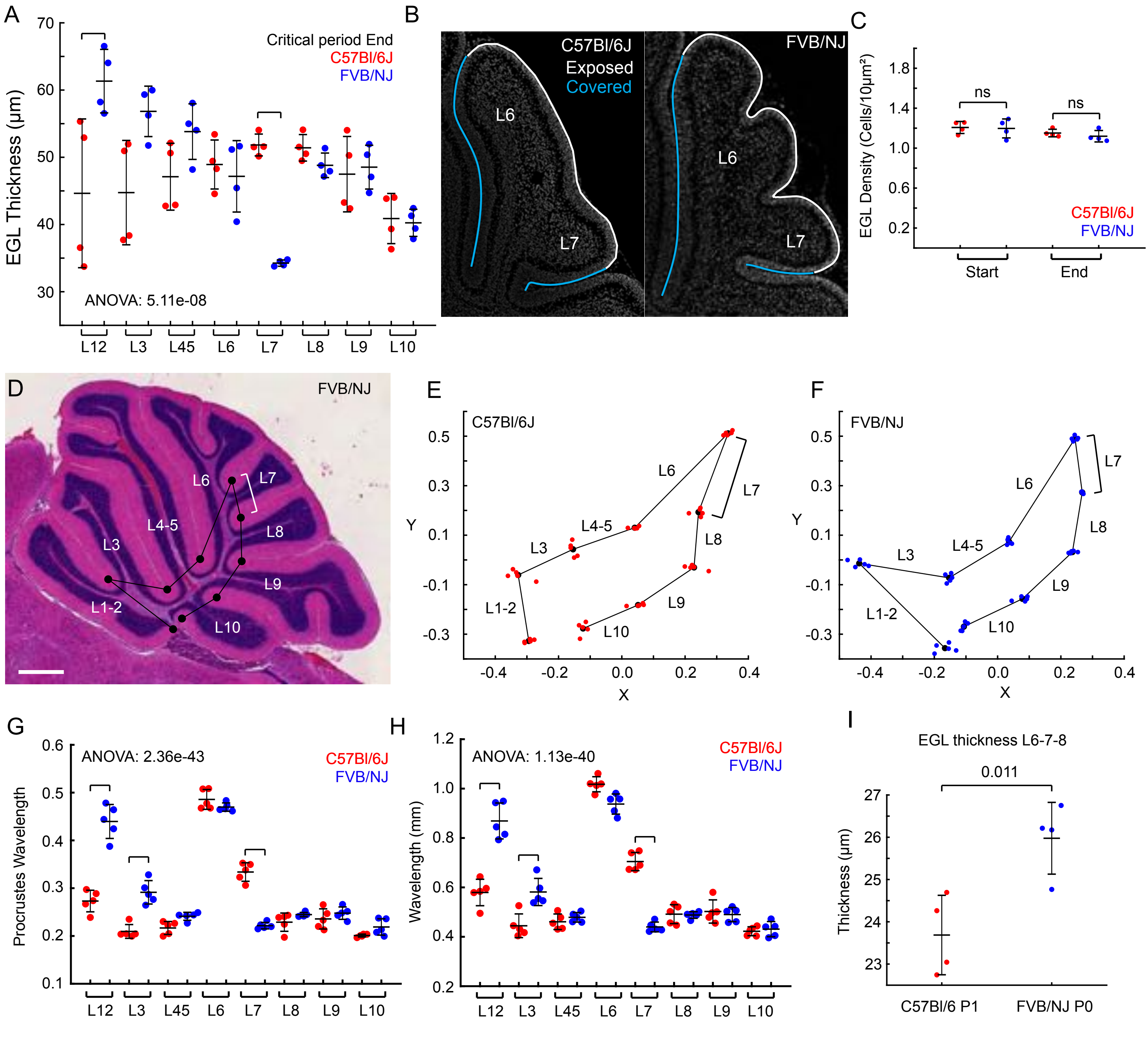
A
Critical period End
C57Bl/6J
FVB/NJ
EGL Thickness (µm)
ANOVA: 5.11e-08
L12
L3
L45
L6
L7
L8
L9
L10
B
C57Bl/6J
Exposed
Covered
FVB/NJ
L6
L7
C
EGL Density (Cells/10µm²)
ns
ns
Start
End
C57Bl/6J
FVB/NJ
D
FVB/NJ
L6
L7
L4-5
L8
L3
L9
L1-2
L10
E
C57Bl/6J
Y
X
F
FVB/NJ
G
ANOVA: 2.36e-43
C57Bl/6J
FVB/NJ
Procrustes Wavelength
H
ANOVA: 1.13e-40
Wavelength (mm)
I
EGL thickness L6-7-8
0.011
Thickness (µm)
C57Bl/6 P1
FVB/NJ P0

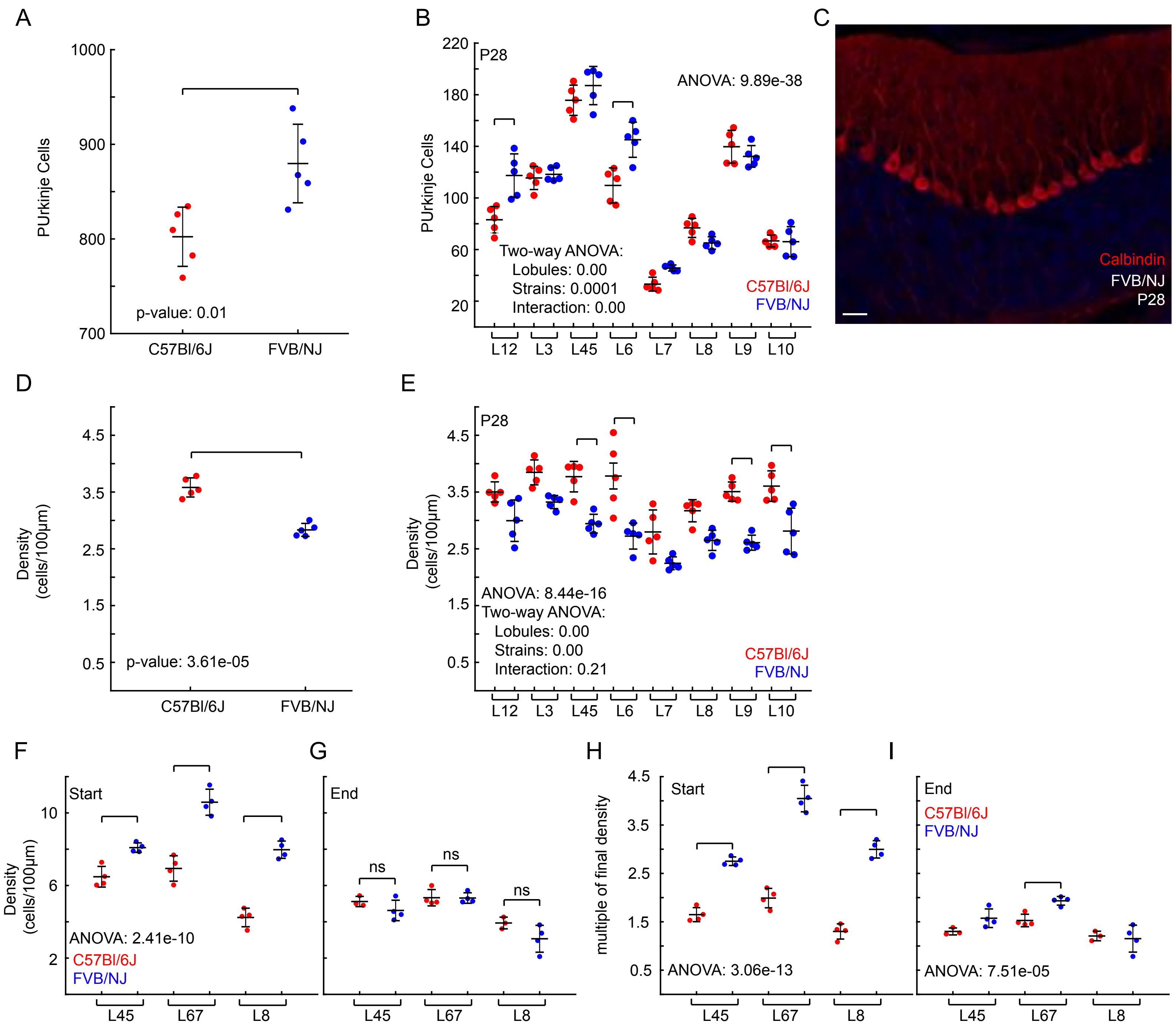
A
PUrkinje Cells
p-value: 0.01
C57Bl/6J
FVB/NJ
B
P28
PUrkinje Cells
ANOVA: 9.89e-38
Two-way ANOVA:
Lobules: 0.00
Strains: 0.0001
Interaction: 0.00
C57Bl/6J
FVB/NJ
L12 L3 L45 L6 L7 L8 L9 L10
C
Calbindin
FVB/NJ
P28
D
Density (cells/100µm)
p-value: 3.61e-05
C57Bl/6J
FVB/NJ
E
P28
Density (cells/100µm)
ANOVA: 8.44e-16
Two-way ANOVA:
Lobules: 0.00
Strains: 0.00
Interaction: 0.21
C57Bl/6J
FVB/NJ
L12 L3 L45 L6 L7 L8 L9 L10
F
Start
Density (cells/100µm)
ANOVA: 2.41e-10
C57Bl/6J
FVB/NJ
L45 L67 L8
G
End
ns
ns
ns
L45 L67 L8
H
Start
multiple of final density
ANOVA: 3.06e-13
L45 L67 L8
I
End
C57Bl/6J
FVB/NJ
ANOVA: 7.51e-05
L45 L67 L8

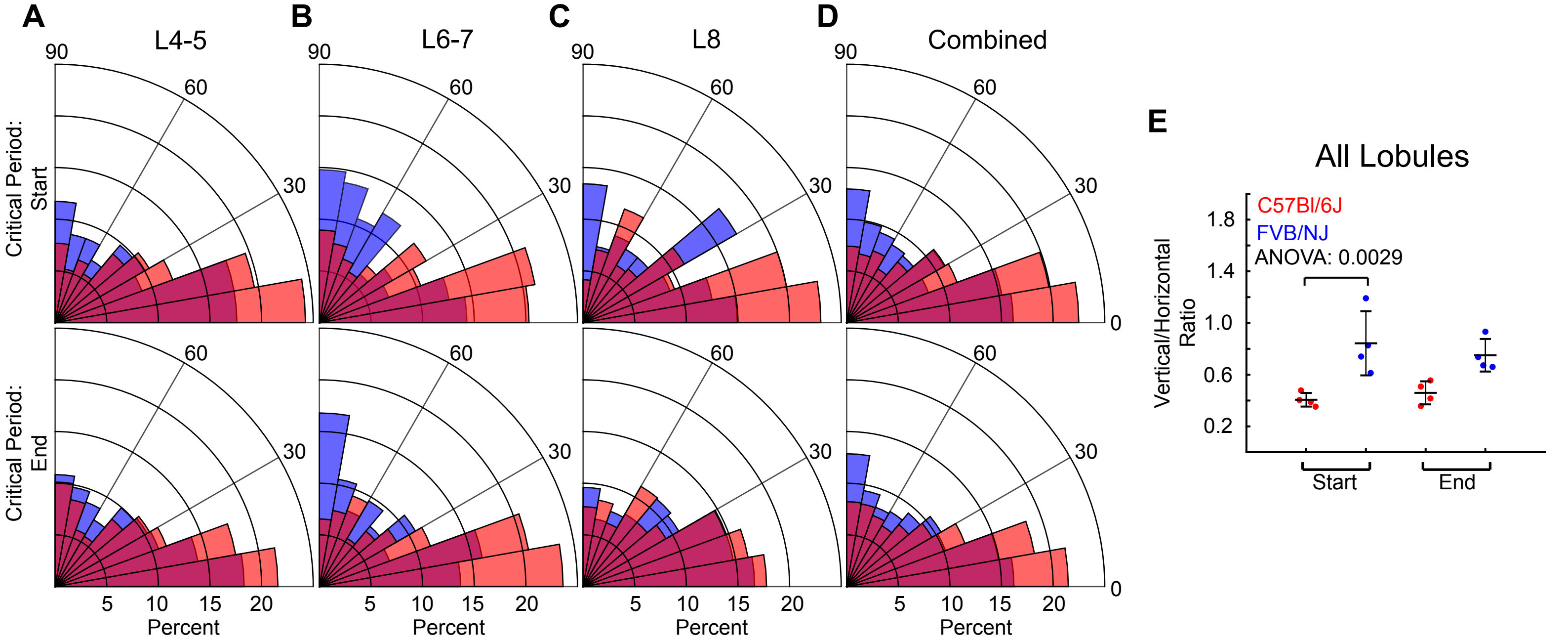

A
B
C
D
E
L4-5
L6-7
L8
Combined
Critical Period: Start
Critical Period: End
90
60
30
0
5
10
15
20
Percent
All Lobules
C57Bl/6J
FVB/NJ
ANOVA: 0.0029
Vertical/Horizontal Ratio
1.8
1.4
1.0
0.6
0.2
Start
End

bioRxiv preprint doi: https://doi.org/10.1101/2023.07.21.549165; this version posted July 21, 2023. The copyright holder for this preprint (which was not certified by peer review) is the author/funder, who has granted bioRxiv a license to display the preprint in perpetuity. It is made available under aCC-BY 4.0 International license.

**Figure 1:**				
	Test	p-value		
1C	ttest2	1.83E-05		
1D	ttest2	4.85E-08		
	Test	p-value	Test	p-value
1E	two-way anova		one-way anova	8.07E-51
	lobules	6.96E-51		
	Strain	6.05E-34		
	Interaction	7.68E-20		
1F	ttest2	7.37E-07		
1G	ttest2	5.23E-09		
**Figure 2:**				
	Test	p-value		
2A	*Non-linear Regression			
2E	ttest2	0.3759		
2F	ttest2	0.0653		
**Figure 3:**				
	Test	p-value		
3A	*Linear Regression			
**Figure 4:**				
	Test	p-value	Test	p-value
4C	two-way anova		one way anova	4.62E-22
	lobules	3.38E-23		
	Strain	8.02E-11		
	Interaction	2.51E-05		
4D	Test	p-value	Test	p-value
	two-way anova		oneway anova	1.138E-11
	lobules	0.00E+00		
	Strain	0.00E+00		
	Interaction	0.4698		
4E	Test	p-value	Test	p-value
	two-way anova		oneway anova	8.972E-05
	lobules	0.00E+00		
	Strain	0.0429		
	Interaction	0.0126		
4F	Test	p-value		
	one-way anova	3.715E-07		
4K	Test	p-value		
	ttest2	1.856E-06		

bioRxiv preprint doi: https://doi.org/10.1101/2023.07.21.549165; this version posted July 21, 2023. The copyright holder for this preprint (which was not certified by peer review) is the author/funder, who has granted bioRxiv a license to display the preprint in perpetuity. It is made available under aCC-BY 4.0 International license.

**Figure 5:**				
5E	Test	p-value		
	one-way anova	4.306E-08		
5H	Test	p-value	Test	p-value
	two-way anova		one-way anova	1.77E-16
	lobules	0.00E+00		
	Strain	0.9944		
	Interaction	0.00E+00		
5I	Test	p-value	Test	p-value
	two-way anova		one-way anova	3.34E-15
	lobules	3.77E-16		
	Strain	7.79E-07		
	Interaction	3.37E-06		
5J	Test	p-value		
	ttest2	0.1665		
5K	Test	p-value		
	ttest2	0.0602		
5O	Test	p-value		
	one-way anova	0.0185		
5P	Test	p-value		
	one-way anova	0.0012		
**Figure 6**				
6F	Test	p-value	Test	p-value
	two-way anova		one-way anova	0.0028
	lobules	0.0626		
	Strain	0.0005		
	Interaction	0.1825		
6G	Test	p-value	Test	p-value
	two-way anova		one-way anova	0.0046
	lobules	0.1378		
	Strain	0.0035		
	Interaction	0.0224		
6H - I	*Linear Regression			

bioRxiv preprint doi: https://doi.org/10.1101/2023.07.21.549165; this version posted July 21, 2023. The copyright holder for this preprint (which was not certified by peer review) is the author/funder, who has granted bioRxiv a license to display the preprint in perpetuity. It is made available under aCC-BY 4.0 International license.

**Supplemental Fig 2**				
2A	Test	p-value		
	ttest2	7.872E-06		
2B	Test	p-value	Test	p-value
	two-way anova		one-way anova	6.60E-51
	lobules	0		
	Strain	0.4711		
	Interaction	0		
2D	Test	p-value	Test	p-value
	two-way anova		one-way anova	4.00E-07
	lobules	0.0005		
	Strain	0		
	Interaction	0.048		
**Supplemental Fig 3**				
3F	Test	p-value		
	ttest2	0.7353		
3G	Test	p-value		
	ttest2	0.9473		
3H	Test	p-value		
	ttest2	0.417		
3I	Test			
	*Linear Regressions			
**Supplemental Fig 4**				
4E	Test	p-value		
	ttest2	0.0044		
4F-I	*Linear Regressions			
**Supplemental Fig 6**				
6A	Test	p-value	Test	p-value
	two-way anova		one-way anova	5.11E-08
	lobules	0.00E+00		
	Strain	0.1547		
	Interaction	0.00E+00		
6C	Test	p-value		
	one-way anova	0.2534		
6G	Test	p-value	Test	p-value
	two-way anova		oneway	2.36E-43
	lobules	3.30E-44		

bioRxiv preprint doi: https://doi.org/10.1101/2023.07.21.549165; this version posted July 21, 2023. The copyright holder for this preprint (which was not certified by peer review) is the author/funder, who has granted bioRxiv a license to display the preprint in perpetuity. It is made available under aCC-BY 4.0 International license.

	Strain	1.06E-07		
	Interaction	1.71E-23		
6H	Test	p-value	Test	p-value
	two-way anova		oneway	1.13E-40
	lobules	0		
	Strain	0.1982		
	Interaction	0		
6I	Test	p-value		
	ttest2	0.0111		
**Supplemental Fig 7**				
7A	Test	p-value		
	ttest2	0.010		
7B	Test	p-value	Test	p-value
	two-way anova		one-way anova	9.89E-38
	lobules	0		
	Strain	0.0001		
	Interaction	0		
7D	Test	p-value		
	ttest2	3.61E-05		
7E	Test	p-value	Test	p-value
	two-way anova		one-way anova	8.44E-16
	lobules	0		
	Strain	0		
	Interaction	0.2054		
7F	Test	p-value	Test	p-value
	two-way anova		one-way anova	2.41E-10
	lobules	0		
	Strain	0		
	Interaction	0.0017		
7G	Test	p-value	Test	p-value
	two-way anova		one-way anova	3.52E-05
	lobules	0		
	Strain	0.0444		
	Interaction	0.2704		
7H	Test	p-value	Test	p-value
	two-way anova		one-way anova	3.06E-13
	lobules	0		
	Strain	0		
	Interaction	0.0002		

bioRxiv preprint doi: https://doi.org/10.1101/2023.07.21.549165; this version posted July 21, 2023. The copyright holder for this preprint (which was not certified by peer review) is the author/funder, who has granted bioRxiv a license to display the preprint in perpetuity. It is made available under aCC-BY 4.0 International license.

7I	Test	p-value	Test	p-value
	two-way anova		one-way anova	7.51E-05
	lobules	0		
	Strain	0.0106		
	Interaction	0.0495		
**Supplemental Fig8**				
8E	Test	p-value		
	one-way anova	0.0029		

bioRxiv preprint doi: https://doi.org/10.1101/2023.07.21.549165; this version posted July 21, 2023. The copyright holder for this preprint (which was not certified by peer review) is the author/funder, who has granted bioRxiv a license to display the preprint in perpetuity. It is made available under aCC-BY 4.0 International license.

Linear Regression Analysis					
Main Figures					
**Figure 3**					
3A	*Multiple Linear Regression*				
	**C57Bl6/J vs FVB/NJ**	Estimate	SE	tStat	pValue
	Intercept	736.91	90.476	8.1447	6.33E-12
	Area	0.003743	0.000171	21.871	2.79E-34
	Strain	-799.26	121.51	-6.5775	5.69E-11
	Interaction	0.001742	0.00023	7.5644	8.02E-11
	Observations	79			
	Deg. Of Freedom	75			
	RMSE	131			
	R-squared	0.959			
	Adj. R-squared	0.957			
	F-stat vs Const.	584			
	p-value	6.71E-52			
**Figure 6**					
6H	*Linear Regression*				
	**CDA vs Expansion**	Estimate	SE	tStat	pValue
	Intercept	226.22	45.036	5.0231	0.00737
	x1	374.56	60.287	6.213	0.003415
	Observations	6			
	Deg. Of Freedom	4			
	RMSE	45.8			
	R-squared	0.906			
	Adj. R-squared	0.883			
	F-stat vs Const.	38.6			
	p-value	0.00342			
6I	*Linear Regression*				
	**CDA vs Thickness**	Estimate	SE	tStat	pValue
	Intercept	56.859	3.257	17.458	8.07E-09
	x1	-14.13	4.7075	-3.0016	0.013308
	Observations	12			
	Deg. Of Freedom	10			
	RMSE	4.24			
	R-squared	0.474			
	Adj. R-squared	0.421			
	F-stat vs Const.	9.01			
	p-value	0.0133			
Supplemental Figures					
**Supplemental Fig 3**					
3I					
	Linear Regression				
	**FVB/NJ**	Estimate	SE	tStat	pValue

bioRxiv preprint doi: https://doi.org/10.1101/2023.07.21.549165; this version posted July 21, 2023. The copyright holder for this preprint (which was not certified by peer review) is the author/funder, who has granted bioRxiv a license to display the preprint in perpetuity. It is made available under aCC-BY 4.0 International license.

	Intercept	0.099801	0.10005	0.99751	0.32536
	x1	4.0451	0.17307	23.373	6.08E-23
	Observations	37			
	Deg. Of Freedom	35			
	RMSE	0.211			
	R-squared	0.94			
	Adj. R-squared	0.938			
	F-stat vs Const.	546			
	p-value	6.08E-23			
	Linear Regression				
	**C57Bl/6J**	Estimate	SE	tStat	pValue
	Intercept	0.06403	0.10781	0.59393	0.55627
	x1	3.9523	0.16637	23.755	1.37E-23
	Observations	38			
	Deg. Of Freedom	36			
	RMSE	0.225			
	R-squared	0.94			
	Adj. R-squared	0.938			
	F-stat vs Const.	564			
	p-value	1.37E-23			
	Multiple Linear Regression				
	**C57Bl6/J vs FVB/NJ**	Estimate	SE	tStat	pValue
	Intercept	0.06403	0.10441	0.61328	0.54165
	Area	3.9523	0.16113	24.529	2.14E-36
	Strain	0.035771	0.14711	0.24315	0.80859
	interaction	0.092779	0.24104	0.38491	0.70146
	Observations	75			
	Deg. Of Freedom	71			
	RMSE	0.218			
	R-squared	0.941			
	Adj. R-squared	0.938			
	F-stat vs Const.	374			
	p-value	1.99E-43			
**Supplemental Fig 4**					
4A	*Multiple Linear Regression*				
	**C57Bl6/J vs FVB/NJ L45**	Estimate	SE	tStat	pValue
	Intercept	863.27	94.726	9.1133	3.13E-13
	Area	0.003891	0.000154	25.301	2.32E-35
	Strain	-403.03	127.89	-3.1513	0.002458
	interaction	0.000983	0.00023	4.275	6.39E-05
	Observations	69			
	Deg. Of Freedom	65			
	RMSE	142			
	R-squared	0.96			
	Adj. R-squared	0.959			

bioRxiv preprint doi: https://doi.org/10.1101/2023.07.21.549165; this version posted July 21, 2023. The copyright holder for this preprint (which was not certified by peer review) is the author/funder, who has granted bioRxiv a license to display the preprint in perpetuity. It is made available under aCC-BY 4.0 International license.

	F-stat vs Const.	525			
	p-value	1.74E-45			
4B	*Multiple Linear Regression*				
	**C57Bl6/J vs FVB/NJ L8**	Estimate	SE	tStat	pValue
	Intercept	517.78	47.879	10.814	4.41E-16
	Area	0.003896	0.00015	25.939	1.14E-35
	Strain	-162.27	59.033	-2.7489	0.007763
	interaction	0.000958	0.000233	4.1063	0.000116
	Observations	68			
	Deg. Of Freedom	64			
	RMSE	70.7			
	R-squared	0.972			
	Adj. R-squared	0.971			
	F-stat vs Const.	736			
	p-value	1.55E-49			
4C	*Multiple Linear Regression*				
	**FVB/NJ L67 vs L45**	Estimate	SE	tStat	pValue
	Intercept	-62.359	88.378	-0.7056	0.48278
	Area	0.005485	0.000168	32.69	3.95E-44
	Lobule	522.6	123.86	4.2191	7.23E-05
	interaction	-0.00061	0.000241	-2.5364	0.013432
	Observations	74			
	Deg. Of Freedom	70			
	RMSE	143			
	R-squared	0.964			
	Adj. R-squared	0.962			
	F-stat vs Const.	623			
	p-value	2.10E-50			
	**FVB/NJ L67 vs L8**	Estimate	SE	tStat	pValue
	Intercept	-62.359	74.239	-0.83998	0.40374
	Area	0.005485	0.000141	38.916	1.26E-49
	Lobule	417.86	94.636	4.4155	3.53E-05
	interaction	-0.00063	0.000334	-1.8862	0.063362
	Observations	75			
	Deg. Of Freedom	71			
	RMSE	120			
	R-squared	0.985			
	Adj. R-squared	0.984			
	F-stat vs Const.	1.55E+03			
	p-value	1.26E-64			
4D	*Multiple Linear Regression*				
	**C57Bl6/J L67 vs L45**	Estimate	SE	tStat	pValue
	Intercept	736.91	88.878	8.2912	5.31E-12
	Area	0.003743	0.000168	22.265	1.75E-33

bioRxiv preprint doi: https://doi.org/10.1101/2023.07.21.549165; this version posted July 21, 2023. The copyright holder for this preprint (which was not certified by peer review) is the author/funder, who has granted bioRxiv a license to display the preprint in perpetuity. It is made available under aCC-BY 4.0 International license.

	Lobule	126.37	123.85	1.0203	0.3111
	interaction	0.000148	0.000219	0.67578	0.5014
	Observations	74			
	Deg. Of Freedom	70			
	RMSE	129			
	R-squared	0.957			
	Adj. R-squared	0.956			
	F-stat vs Const.	524			
	p-value	7.12E-48			
	**C57Bl6/J L67 vs L8**	Estimate	SE	tStat	pValue
	Intercept	736.91	64.059	11.504	1.31E-17
	Area	0.003743	0.000121	30.891	9.52E-42
	Lobule	-219.13	89.813	-2.4398	0.017308
	interaction	0.000153	0.000232	0.66106	0.51081
	Observations	72			
	Deg. Of Freedom	68			
	RMSE	93			
	R-squared	0.979			
	Adj. R-squared	0.978			
	F-stat vs Const.	1.06E+03			
	p-value	5.32E-57			
4E	*Linear Regression*				
	**FVB/NJ L45**	Estimate	SE	tStat	pValue
	Intercept	460.24	81.961	5.6154	3.68E-06
	x1	0.004874	0.000163	29.892	2.01E-24
	Observations	33			
	Deg. Of Freedom	31			
	RMSE	135			
	R-squared	0.966			
	Adj. R-squared	0.965			
	F-stat vs Const.	894			
	p-value	2.01E-24			
	Linear Regression				
	**FVB/NJ L67**	Estimate	SE	tStat	pValue
	Intercept	-62.359	92.097	-0.6771	0.50234
	x1	0.005485	0.000175	31.37	2.78E-29
	Observations	41			
	Deg. Of Freedom	39			
	RMSE	149			
	R-squared	0.962			
	Adj. R-squared	0.961			
	F-stat vs Const.	984			
	p-value	2.78E-29			
	Linear Regression				

bioRxiv preprint doi: https://doi.org/10.1101/2023.07.21.549165; this version posted July 21, 2023. The copyright holder for this preprint (which was not certified by peer review) is the author/funder, who has granted bioRxiv a license to display the preprint in perpetuity. It is made available under aCC-BY 4.0 International license.

	**FVB/NJ L8**	Estimate	SE	tStat	pValue
	Intercept	355.51	34.38	10.34	9.93E-12
	x1	0.004854	0.000178	27.329	9.41E-24
	Observations	34			
	Deg. Of Freedom	32			
	RMSE	70.4			
	R-squared	0.959			
	Adj. R-squared	0.958			
	F-stat vs Const.	747			
	p-value	9.41E-24			
	**C57Bl/6J L45**	Estimate	SE	tStat	pValue
	Intercept	863.27	98.546	8.7601	3.09E-10
	x1	0.003891	0.00016	24.32	4.40E-23
	Observations	36			
	Deg. Of Freedom	34			
	RMSE	147			
	R-squared	0.946			
	Adj. R-squared	0.944			
	F-stat vs Const.	591			
	p-value	4.40E-23			
	**C57Bl/6J L67**	Estimate	SE	tStat	pValue
	Intercept	736.91	74.984	9.8275	9.87E-12
	x1	0.003743	0.000142	26.39	3.79E-25
	Observations	38			
	Deg. Of Freedom	36			
	RMSE	109			
	R-squared	0.951			
	Adj. R-squared	0.949			
	F-stat vs Const.	696			
	p-value	3.79E-25			
	**C57Bl/6J L8**	Estimate	SE	tStat	pValue
	Intercept	517.78	48.089	10.767	3.61E-12
	x1	0.003896	0.000151	25.825	5.33E-23
	Observations	34			
	Deg. Of Freedom	32			
	RMSE	71			
	R-squared	0.954			
	Adj. R-squared	0.953			
	F-stat vs Const.	667			
	p-value	5.33E-23			

bioRxiv preprint doi: https://doi.org/10.1101/2023.07.21.549165; this version posted July 21, 2023. The copyright holder for this preprint (which was not certified by peer review) is the author/funder, who has granted bioRxiv a license to display the preprint in perpetuity. It is made available under aCC-BY 4.0 International license.

Non-Linear Regression Analysis						
Figure 2						
2A	Global Data					
	Non Linear Regression (Gompertz)					
	**FVB/NJ Fit**	Estimate	SE	tStat	pValue	
	beta1	26.041	0.4991	52.175	1.14E-70	
	beta2	3.4008	0.070694	48.107	1.65E-67	
	beta3	0.64528	0.018903	34.137	2.12E-54	
	Observations	96				
	Deg. Of Freedom	93				
	RMSE	0.64				
	R-squared	0.991	*not applicable for non-linear regressions			
	Adj. R-squared	0.991	*not applicable for non-linear regressions			
	F-stat vs Const.	9.40E+03				
	p-value	2.64E-115				
	**C57Bl/6J Fit**	Estimate	SE	tStat	pValue	
	beta1	21.371	0.33822	63.188	5.74E-77	
	beta2	3.5276	0.070256	50.211	3.91E-68	
	beta3	0.7795	0.021169	36.822	1.87E-56	
	Observations	94				
	Deg. Of Freedom	91				
	RMSE	0.463				
	R-squared	0.994	*not applicable for non-linear regressions			
	Adj. R-squared	0.994	*not applicable for non-linear regressions			
	F-stat vs Const.	1.63E+04				
	p-value	4.22E-124				
	**Combined Fit**	Estimate	SE	tStat	pValue	
	beta1	24.254	0.4001	60.621	6.72E-125	
	beta2	3.3912	0.057778	58.693	2.10E-122	
	beta3	0.67623	0.01709	39.569	8.32E-93	
	Observations	190				
	Deg. Of Freedom	187				
	RMSE	0.686				
	R-squared	0.988	*not applicable for non-linear regressions			
	Adj. R-squared	0.988	*not applicable for non-linear regressions			
	F-stat vs Const.	1.56E+04				
	p-value	4.20E-224				
